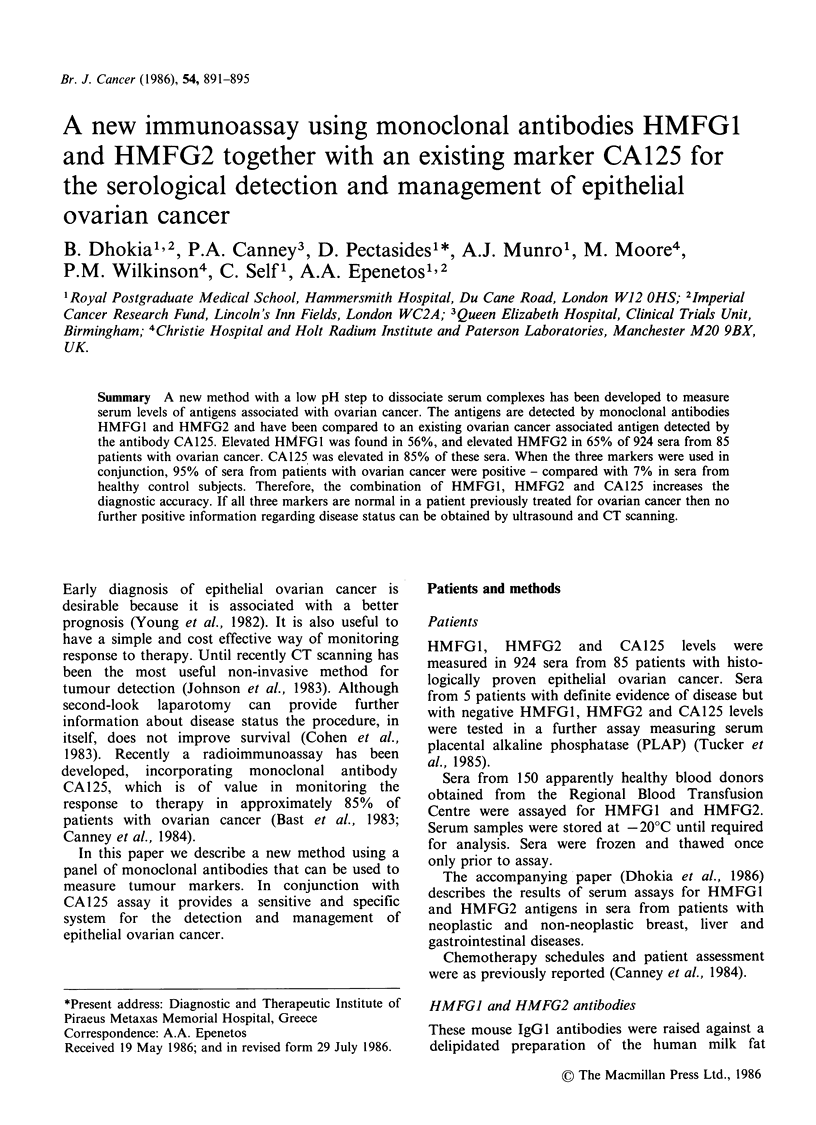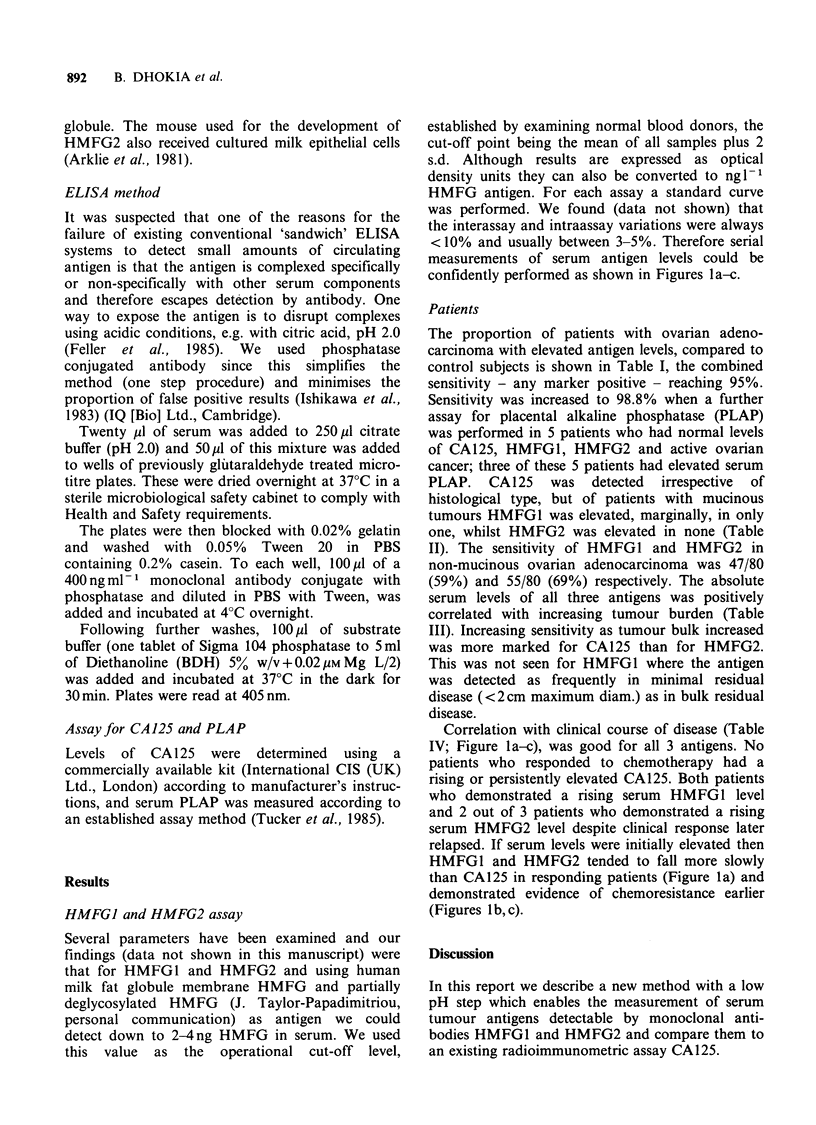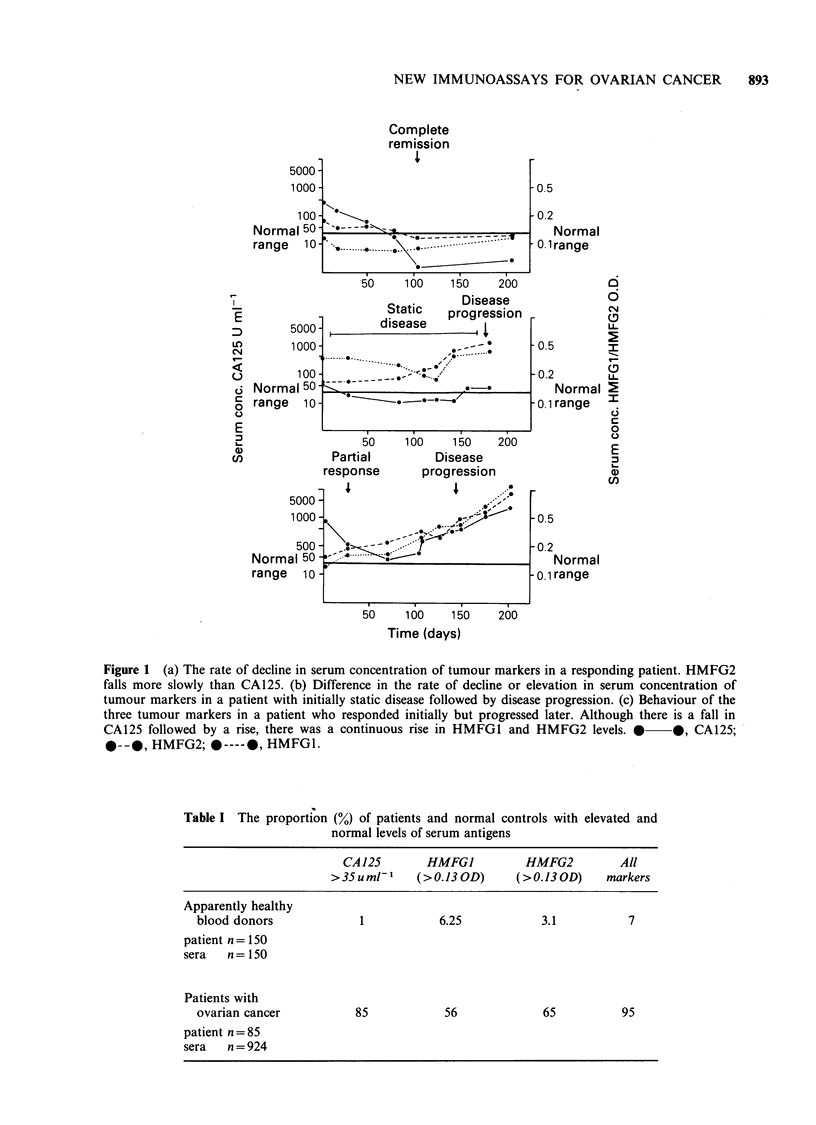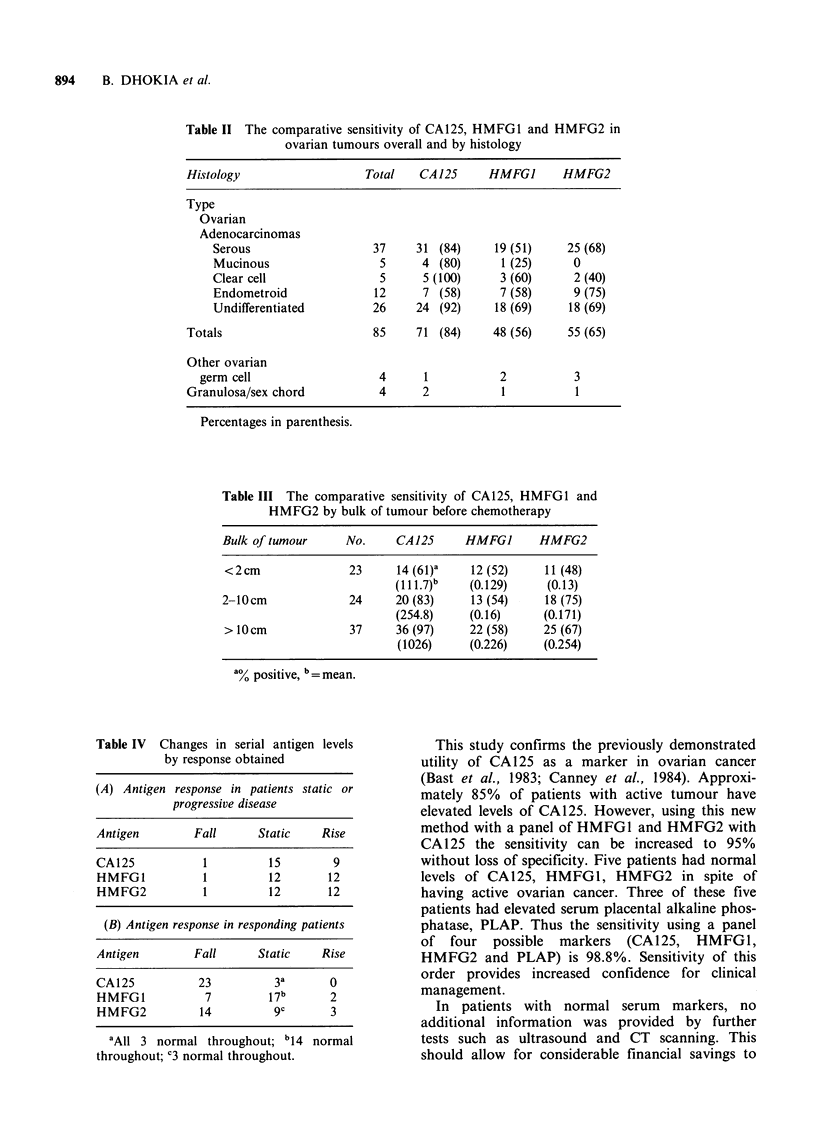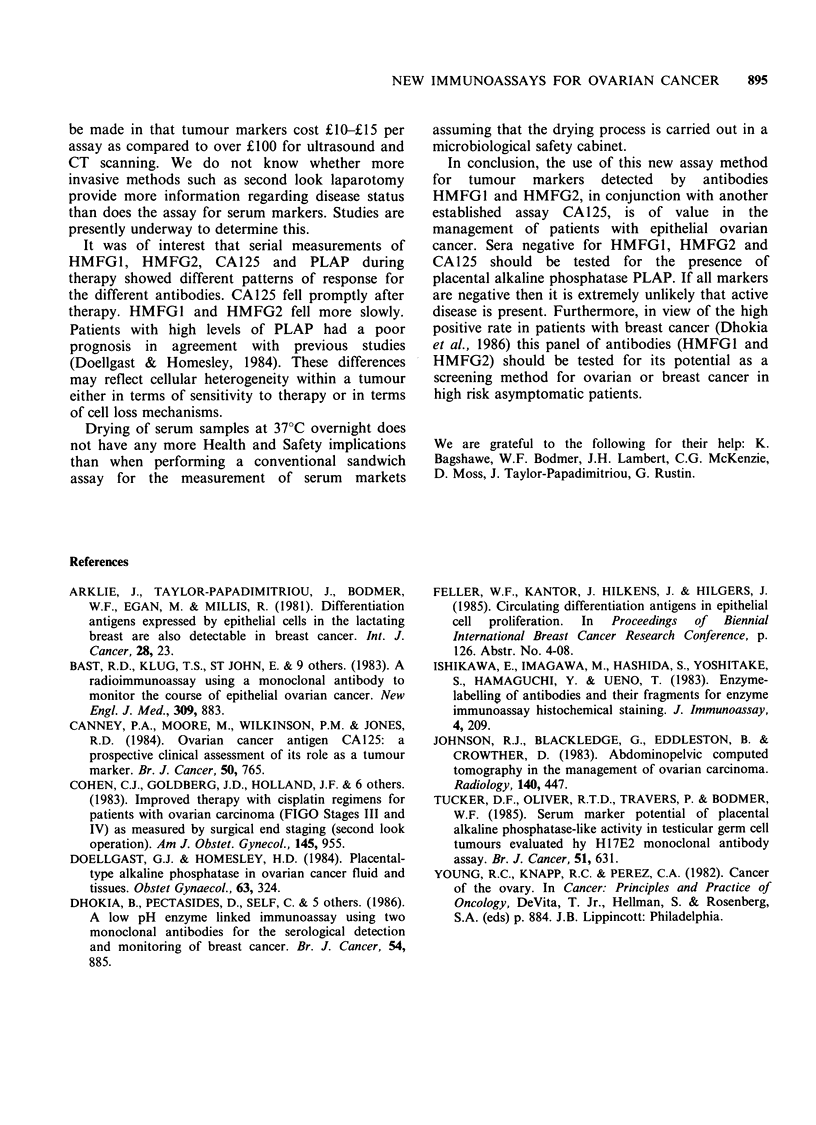# A new immunoassay using monoclonal antibodies HMFG1 and HMFG2 together with an existing marker CA125 for the serological detection and management of epithelial ovarian cancer.

**DOI:** 10.1038/bjc.1986.258

**Published:** 1986-12

**Authors:** B. Dhokia, P. A. Canney, D. Pectasides, A. J. Munro, M. Moore, P. M. Wilkinson, C. Self, A. A. Epenetos

## Abstract

A new method with a low pH step to dissociate serum complexes has been developed to measure serum levels of antigens associated with ovarian cancer. The antigens are detected by monoclonal antibodies HMFG1 and HMFG2 and have been compared to an existing ovarian cancer associated antigen detected by the antibody CA125. Elevated HMFG1 was found in 56%, and elevated HMFG2 in 65% of 924 sera from 85 patients with ovarian cancer. CA125 was elevated in 85% of these sera. When the three markers were used in conjunction, 95% of sera from patients with ovarian cancer were positive--compared with 7% in sera from healthy control subjects. Therefore, the combination of HMFG1, HMFG2 and CA125 increases the diagnostic accuracy. If all three markers are normal in a patient previously treated for ovarian cancer then no further positive information regarding disease status can be obtained by ultrasound and CT scanning.


*Br. J. Cancer* (1986), **54**, 891–895

### A new immunoassay using monoclonal antibodies HMFG1 and HMFG2 together with an existing marker CA125 for the serological detection and management of epithelial ovarian cancer

B. Dhokia[1,2], P.A. Canney[3], D. Pectasides[1]*, A.J. Munro[1], M. Moore[4], P.M. Wilkinson[4], C. Self[1], A.A. Epenetos[1,2]

[1]*Royal Postgraduate Medical School, Hammersmith Hospital, Du Cane Road, London W12 0HS;* [2]*Imperial Cancer Research Fund, Lincoln's Inn Fields, London WC2A;* [3]*Queen Elizabeth Hospital, Clinical Trials Unit, Birmingham;* [4]*Christie Hospital and Holt Radium Institute and Paterson Laboratories, Manchester M20 9BX, UK.*

**Summary** A new method with a low pH step to dissociate serum complexes has been developed to measure serum levels of antigens associated with ovarian cancer. The antigens are detected by monoclonal antibodies HMFG1 and HMFG2 and have been compared to an existing ovarian cancer associated antigen detected by the antibody CA125. Elevated HMFG1 was found in 56%, and elevated HMFG2 in 65% of 924 sera from 85 patients with ovarian cancer. CA125 was elevated in 85% of these sera. When the three markers were used in conjunction, 95% of sera from patients with ovarian cancer were positive – compared with 7% in sera from healthy control subjects. Therefore, the combination of HMFG1, HMFG2 and CA125 increases the diagnostic accuracy. If all three markers are normal in a patient previously treated for ovarian cancer then no further positive information regarding disease status can be obtained by ultrasound and CT scanning.

Early diagnosis of epithelial ovarian cancer is desirable because it is associated with a better prognosis (Young *et al.*, 1982). It is also useful to have a simple and cost effective way of monitoring response to therapy. Until recently CT scanning has been the most useful non-invasive method for tumour detection (Johnson *et al.*, 1983). Although second-look laparotomy can provide further information about disease status the procedure, in itself, does not improve survival (Cohen *et al.*, 1983). Recently a radioimmunoassay has been developed, incorporating monoclonal antibody CA125, which is of value in monitoring the response to therapy in approximately 85% of patients with ovarian cancer (Bast *et al.*, 1983; Canney *et al.*, 1984).

In this paper we describe a new method using a panel of monoclonal antibodies that can be used to measure tumour markers. In conjunction with CA125 assay it provides a sensitive and specific system for the detection and management of epithelial ovarian cancer.

*Present address: Diagnostic and Therapeutic Institute of Piraeus Metaxas Memorial Hospital, Greece
Correspondence: A.A. Epenetos
Received 19 May 1986; and in revised form 29 July 1986.

### Patients and methods

### *Patients*

HMFG1, HMFG2 and CA125 levels were measured in 924 sera from 85 patients with histologically proven epithelial ovarian cancer. Sera from 5 patients with definite evidence of disease but with negative HMFG1, HMFG2 and CA125 levels were tested in a further assay measuring serum placental alkaline phosphatase (PLAP) (Tucker *et al.*, 1985).

Sera from 150 apparently healthy blood donors obtained from the Regional Blood Transfusion Centre were assayed for HMFG1 and HMFG2. Serum samples were stored at −20°C until required for analysis. Sera were frozen and thawed once only prior to assay.

The accompanying paper (Dhokia *et al.*, 1986) describes the results of serum assays for HMFG1 and HMFG2 antigens in sera from patients with neoplastic and non-neoplastic breast, liver and gastrointestinal diseases.

Chemotherapy schedules and patient assessment were as previously reported (Canney *et al.*, 1984).

### *HMFG1 and HMFG2 antibodies*

These mouse IgG1 antibodies were raised against a delipidated preparation of the human milk fat

© The Macmillan Press Ltd., 1986

globule. The mouse used for the development of HMFG2 also received cultured milk epithelial cells (Arklie et al., 1981).

*ELISA method*

It was suspected that one of the reasons for the failure of existing conventional 'sandwich' ELISA systems to detect small amounts of circulating antigen is that the antigen is complexed specifically or non-specifically with other serum components and therefore escapes detection by antibody. One way to expose the antigen is to disrupt complexes using acidic conditions, e.g. with citric acid, pH 2.0 (Feller et al., 1985). We used phosphatase conjugated antibody since this simplifies the method (one step procedure) and minimises the proportion of false positive results (Ishikawa et al., 1983) (IQ [Bio] Ltd., Cambridge).

Twenty $\mu$l of serum was added to 250 $\mu$l citrate buffer (pH 2.0) and 50 $\mu$l of this mixture was added to wells of previously glutaraldehyde treated microtitre plates. These were dried overnight at 37°C in a sterile microbiological safety cabinet to comply with Health and Safety requirements.

The plates were then blocked with 0.02% gelatin and washed with 0.05% Tween 20 in PBS containing 0.2% casein. To each well, 100 $\mu$l of a 400 ng ml$^{-1}$ monoclonal antibody conjugate with phosphatase and diluted in PBS with Tween, was added and incubated at 4°C overnight.

Following further washes, 100 $\mu$l of substrate buffer (one tablet of Sigma 104 phosphatase to 5 ml of Diethanoline (BDH) 5% w/v + 0.02 $\mu$M Mg L/2) was added and incubated at 37°C in the dark for 30 min. Plates were read at 405 nm.

*Assay for CA125 and PLAP*

Levels of CA125 were determined using a commercially available kit (International CIS (UK) Ltd., London) according to manufacturer's instructions, and serum PLAP was measured according to an established assay method (Tucker et al., 1985).

### Results

*HMFG1 and HMFG2 assay*

Several parameters have been examined and our findings (data not shown in this manuscript) were that for HMFG1 and HMFG2 and using human milk fat globule membrane HMFG and partially deglycosylated HMFG (J. Taylor-Papadimitriou, personal communication) as antigen we could detect down to 2–4 ng HMFG in serum. We used this value as the operational cut-off level, established by examining normal blood donors, the cut-off point being the mean of all samples plus 2 s.d. Although results are expressed as optical density units they can also be converted to ng l$^{-1}$ HMFG antigen. For each assay a standard curve was performed. We found (data not shown) that the interassay and intraassay variations were always <10% and usually between 3–5%. Therefore serial measurements of serum antigen levels could be confidently performed as shown in Figures 1a–c.

*Patients*

The proportion of patients with ovarian adenocarcinoma with elevated antigen levels, compared to control subjects is shown in Table I, the combined sensitivity – any marker positive – reaching 95%. Sensitivity was increased to 98.8% when a further assay for placental alkaline phosphatase (PLAP) was performed in 5 patients who had normal levels of CA125, HMFG1, HMFG2 and active ovarian cancer; three of these 5 patients had elevated serum PLAP. CA125 was detected irrespective of histological type, but of patients with mucinous tumours HMFG1 was elevated, marginally, in only one, whilst HMFG2 was elevated in none (Table II). The sensitivity of HMFG1 and HMFG2 in non-mucinous ovarian adenocarcinoma was 47/80 (59%) and 55/80 (69%) respectively. The absolute serum levels of all three antigens was positively correlated with increasing tumour burden (Table III). Increasing sensitivity as tumour bulk increased was more marked for CA125 than for HMFG2. This was not seen for HMFG1 where the antigen was detected as frequently in minimal residual disease (<2 cm maximum diam.) as in bulk residual disease.

Correlation with clinical course of disease (Table IV; Figure 1a–c), was good for all 3 antigens. No patients who responded to chemotherapy had a rising or persistently elevated CA125. Both patients who demonstrated a rising serum HMFG1 level and 2 out of 3 patients who demonstrated a rising serum HMFG2 level despite clinical response later relapsed. If serum levels were initially elevated then HMFG1 and HMFG2 tended to fall more slowly than CA125 in responding patients (Figure 1a) and demonstrated evidence of chemoresistance earlier (Figures 1b, c).

### Discussion

In this report we describe a new method with a low pH step which enables the measurement of serum tumour antigens detectable by monoclonal antibodies HMFG1 and HMFG2 and compare them to an existing radioimmunometric assay CA125.

**Figure 1** (a) The rate of decline in serum concentration of tumour markers in a responding patient. HMFG2 falls more slowly than CA125. (b) Difference in the rate of decline or elevation in serum concentration of tumour markers in a patient with initially static disease followed by disease progression. (c) Behaviour of the three tumour markers in a patient who responded initially but progressed later. Although there is a fall in CA125 followed by a rise, there was a continuous rise in HMFG1 and HMFG2 levels. ●——●, CA125; ●--●, HMFG2; ●----●, HMFG1.

**Table I** The proportion (%) of patients and normal controls with elevated and normal levels of serum antigens

| | *CA125* $>35\,u\,ml^{-1}$ | *HMFG1* (>*0.13 OD*) | *HMFG2* (>*0.13 OD*) | *All markers* |
|---|---|---|---|---|
| Apparently healthy blood donors | 1 | 6.25 | 3.1 | 7 |
| patient $n = 150$<br>sera $n = 150$ | | | | |
| Patients with ovarian cancer | 85 | 56 | 65 | 95 |
| patient $n = 85$<br>sera $n = 924$ | | | | |

**Table II** The comparative sensitivity of CA125, HMFG1 and HMFG2 in ovarian tumours overall and by histology

| *Histology* | *Total* | *CA125* | *HMFG1* | *HMFG2* |
|---|---|---|---|---|
| Type | | | | |
| Ovarian | | | | |
| Adenocarcinomas | | | | |
| Serous | 37 | 31 (84) | 19 (51) | 25 (68) |
| Mucinous | 5 | 4 (80) | 1 (25) | 0 |
| Clear cell | 5 | 5 (100) | 3 (60) | 2 (40) |
| Endometroid | 12 | 7 (58) | 7 (58) | 9 (75) |
| Undifferentiated | 26 | 24 (92) | 18 (69) | 18 (69) |
| Totals | 85 | 71 (84) | 48 (56) | 55 (65) |
| Other ovarian | | | | |
| germ cell | 4 | 1 | 2 | 3 |
| Granulosa/sex chord | 4 | 2 | 1 | 1 |

Percentages in parenthesis.

**Table III** The comparative sensitivity of CA125, HMFG1 and HMFG2 by bulk of tumour before chemotherapy

| *Bulk of tumour* | *No.* | *CA125* | *HMFG1* | *HMFG2* |
|---|---|---|---|---|
| <2 cm | 23 | 14 (61)[a] | 12 (52) | 11 (48) |
| | | (111.7)[b] | (0.129) | (0.13) |
| 2–10 cm | 24 | 20 (83) | 13 (54) | 18 (75) |
| | | (254.8) | (0.16) | (0.171) |
| >10 cm | 37 | 36 (97) | 22 (58) | 25 (67) |
| | | (1026) | (0.226) | (0.254) |

[a]% positive, [b] = mean.

**Table IV** Changes in serial antigen levels by response obtained

*(A) Antigen response in patients static or progressive disease*

| *Antigen* | *Fall* | *Static* | *Rise* |
|---|---|---|---|
| CA125 | 1 | 15 | 9 |
| HMFG1 | 1 | 12 | 12 |
| HMFG2 | 1 | 12 | 12 |

*(B) Antigen response in responding patients*

| *Antigen* | *Fall* | *Static* | *Rise* |
|---|---|---|---|
| CA125 | 23 | 3[a] | 0 |
| HMFG1 | 7 | 17[b] | 2 |
| HMFG2 | 14 | 9[c] | 3 |

[a]All 3 normal throughout; [b]14 normal throughout; [c]3 normal throughout.

This study confirms the previously demonstrated utility of CA125 as a marker in ovarian cancer (Bast *et al.*, 1983; Canney *et al.*, 1984). Approximately 85% of patients with active tumour have elevated levels of CA125. However, using this new method with a panel of HMFG1 and HMFG2 with CA125 the sensitivity can be increased to 95% without loss of specificity. Five patients had normal levels of CA125, HMFG1, HMFG2 in spite of having active ovarian cancer. Three of these five patients had elevated serum placental alkaline phosphatase, PLAP. Thus the sensitivity using a panel of four possible markers (CA125, HMFG1, HMFG2 and PLAP) is 98.8%. Sensitivity of this order provides increased confidence for clinical management.

In patients with normal serum markers, no additional information was provided by further tests such as ultrasound and CT scanning. This should allow for considerable financial savings to be made in that tumour markers cost £10–£15 per assay as compared to over £100 for ultrasound and CT scanning. We do not know whether more invasive methods such as second look laparotomy provide more information regarding disease status than does the assay for serum markers. Studies are presently underway to determine this.

It was of interest that serial measurements of HMFG1, HMFG2, CA125 and PLAP during therapy showed different patterns of response for the different antibodies. CA125 fell promptly after therapy. HMFG1 and HMFG2 fell more slowly. Patients with high levels of PLAP had a poor prognosis in agreement with previous studies (Doellgast & Homesley, 1984). These differences may reflect cellular heterogeneity within a tumour either in terms of sensitivity to therapy or in terms of cell loss mechanisms.

Drying of serum samples at 37°C overnight does not have any more Health and Safety implications than when performing a conventional sandwich assay for the measurement of serum markets assuming that the drying process is carried out in a microbiological safety cabinet.

In conclusion, the use of this new assay method for tumour markers detected by antibodies HMFG1 and HMFG2, in conjunction with another established assay CA125, is of value in the management of patients with epithelial ovarian cancer. Sera negative for HMFG1, HMFG2 and CA125 should be tested for the presence of placental alkaline phosphatase PLAP. If all markers are negative then it is extremely unlikely that active disease is present. Furthermore, in view of the high positive rate in patients with breast cancer (Dhokia *et al.*, 1986) this panel of antibodies (HMFG1 and HMFG2) should be tested for its potential as a screening method for ovarian or breast cancer in high risk asymptomatic patients.

We are grateful to the following for their help: K. Bagshawe, W.F. Bodmer, J.H. Lambert, C.G. McKenzie, D. Moss, J. Taylor-Papadimitriou, G. Rustin.

### References

ARKLIE, J., TAYLOR-PAPADIMITRIOU, J., BODMER, W.F., EGAN, M. & MILLIS, R. (1981). Differentiation antigens expressed by epithelial cells in the lactating breast are also detectable in breast cancer. *Int. J. Cancer*, **28**, 23.

BAST, R.D., KLUG, T.S., ST JOHN, E. & 9 others. (1983). A radioimmunoassay using a monoclonal antibody to monitor the course of epithelial ovarian cancer. *New Engl. J. Med.*, **309**, 883.

CANNEY, P.A., MOORE, M., WILKINSON, P.M. & JONES, R.D. (1984). Ovarian cancer antigen CA125: a prospective clinical assessment of its role as a tumour marker. *Br. J. Cancer*, **50**, 765.

COHEN, C.J., GOLDBERG, J.D., HOLLAND, J.F. & 6 others. (1983). Improved therapy with cisplatin regimens for patients with ovarian carcinoma (FIGO Stages III and IV) as measured by surgical end staging (second look operation). *Am J. Obstet. Gynecol.*, **145**, 955.

DOELLGAST, G.J. & HOMESLEY, H.D. (1984). Placental-type alkaline phosphatase in ovarian cancer fluid and tissues. *Obstet Gynaecol.*, **63**, 324.

DHOKIA, B., PECTASIDES, D., SELF, C. & 5 others. (1986). A low pH enzyme linked immunoassay using two monoclonal antibodies for the serological detection and monitoring of breast cancer. *Br. J. Cancer*, **54**, 885.

FELLER, W.F., KANTOR, J. HILKENS, J. & HILGERS, J. (1985). Circulating differentiation antigens in epithelial cell proliferation. In *Proceedings of Biennial International Breast Cancer Research Conference*, p. 126. Abstr. No. 4-08.

ISHIKAWA, E., IMAGAWA, M., HASHIDA, S., YOSHITAKE, S., HAMAGUCHI, Y. & UENO, T. (1983). Enzyme-labelling of antibodies and their fragments for enzyme immunoassay histochemical staining. *J. Immunoassay*, **4**, 209.

JOHNSON, R.J., BLACKLEDGE, G., EDDLESTON, B. & CROWTHER, D. (1983). Abdominopelvic computed tomography in the management of ovarian carcinoma. *Radiology*, **140**, 447.

TUCKER, D.F., OLIVER, R.T.D., TRAVERS, P. & BODMER, W.F. (1985). Serum marker potential of placental alkaline phosphatase-like activity in testicular germ cell tumours evaluated hy H17E2 monoclonal antibody assay. *Br. J. Cancer*, **51**, 631.

YOUNG, R.C., KNAPP, R.C. & PEREZ, C.A. (1982). Cancer of the ovary. In *Cancer: Principles and Practice of Oncology*, DeVita, T. Jr., Hellman, S. & Rosenberg, S.A. (eds) p. 884. J.B. Lippincott: Philadelphia.